# Frequency and Outcomes of Ipsilateral Axillary Lymphadenopathy After COVID-19 Vaccination

**DOI:** 10.1001/jamanetworkopen.2022.16172

**Published:** 2022-06-08

**Authors:** Wenhui Zhou, Wendy B. DeMartini, Debra M. Ikeda

**Affiliations:** 1 Breast Imaging Division, Department of Radiology, Stanford University Medical Center, Stanford, California

## Abstract

This case series reports on the frequency and outcomes of breast imaging–identified ipsilateral axillary lymphadenopathy after recent COVID-19 vaccination among women.

## Introduction

Ipsilateral axillary lymphadenopathy (IAL) after COVID-19 vaccination results in diagnostic conundrums.^1,2,3^ Expert guidelines advise IAL management based on patient symptoms, imaging results, and risk factors.^2,4,5^ We report on the frequency and outcomes of breast imaging–identified IAL after recent COVID-19 vaccination.

## Methods

In this single-center study, we searched radiology reports from March 19 to October 30, 2021, to identify women with recent COVID-19 vaccinations (≤6 weeks) and breast imaging–detected IAL, recording patient risk factors, symptoms, vaccination date, and vaccine manufacturer. Aligning with guidelines at the time,^2,4,6^ we managed IAL based on patient factors as follows: (1) current ipsilateral suspicious imaging finding or ipsilateral breast cancer, lung cancer, melanoma, or other regional cancer with immediate ultrasonography or biopsy; (2) history of ipsilateral breast cancer with 8-week follow-up ultrasonography; or (3) no risk factors or normal results of ipsilateral breast imaging with clinical follow-up with referral for ultrasonography if clinical signs or symptoms are present for more than 8 weeks. We tabulated IAL frequency, follow-up imaging results, and outcomes. The Pearson χ^2^ test was used to compare categorical variables, and the *t* test was used to compare continuous variables. All statistical tests reported were 2-tailed, and *P* < .05 was considered statistically significant. The Stanford University institutional review board approved this Health Insurance Portability and Accountability Act–compliant study with waiver of informed consent. This study followed the reporting guideline for case series.

## Results

As shown in the Table and Figure, 3008 of 15 468 patients (19%) who underwent imaging had received recent COVID-19 vaccinations. Of 3008 women, 308 (10%) (mean [SD] age, 53 [12] years) had postvaccination IAL detected on screening mammograms (156 of 1834 patients who underwent screening mammograms [8%]), diagnostic mammograms (72 of 883 patients who underwent diagnostic mammograms [8%]), or magnetic resonance imaging scan (80 of 291 patients who underwent magnetic resonance imaging scan [27%]). The frequency of IAL detections by vaccine manufacturer was 9% (172 of 1836) for BioNTech-Pfizer, 12% (126 of 1045) for Moderna, and 5% (7 of 127) for Johnson & Johnson (the manufacturer was unknown or not reported for 3 patients). Of 308 patients with postvaccination IAL, 259 (84%) without risk factors had clinical follow-up and were not referred for follow-up imaging.

**Table.** Risk-Stratified Patient Demographic Characteristics and Diagnostic Evaluation of Ipsilateral Axillary Lymphadenopathy After COVID-19 Vaccination

Characteristic	Patients, No. (%)		*P* value
	Low risk (n = 272)	High risk (n = 36)	
Age, mean (SD), y	53 (12)	53 (16)	.64
Risk factors			
Prior ipsilateral breast cancer	0	23 (64)	NA
Suspicious ipsilateral abnormality	0	3 (8)	
Concurrent ipsilateral breast cancer	0	4 (11)	
Concurrent ipsilateral regional malignant neoplasm	0	6 (17)	
Vaccine manufacturer			
BioNTech-Pfizer	153 (56)	19 (53)	.64
Moderna	110 (40)	16 (44)	
Johnson & Johnson	6 (2)	1 (3)	
Unknown or not reported	3 (1)	0	
Time interval to initial imaging, mean (SD), wk	4 (2)	4 (2)	.49
Screening mammogram	153 (56)	3 (8)	<.001
Diagnostic mammogram	52 (19)	20 (56)	
Breast magnetic resonance imaging	67 (25)	13 (36)	
Warranted follow-up ultrasonography, No./total No. (%)	13/272 (5)	36/36 (100)	NA
Compliance with follow-up	13/13 (100)	34/36 (94)	NA
Time interval to follow-up imaging, mean (SD), wk	15 (8)	8 (4)	.001
Resolution of IAL on imaging	7/13 (54)	22/34 (65)	.49
BI-RADS assessment, No./total No. (%)			
2	7/13 (54)	22/34 (65)	.73
3	2/13 (15)	3/34 (9)	
4	4/13 (31)	9/34 (26)	
5	0/13 (0)	0/34 (0)	
Biopsy workup results, No./total No. (%)			
Benign	5/5 (100)	12/12 (100)	NA
Malignant	0/5 (0)	0/12 (0)	NA

Abbreviations: BI-RADS, Breast Imaging–Reporting and Data System; IAL, ipsilateral axillary lymphadenopathy; NA, not applicable.

**Figure.**
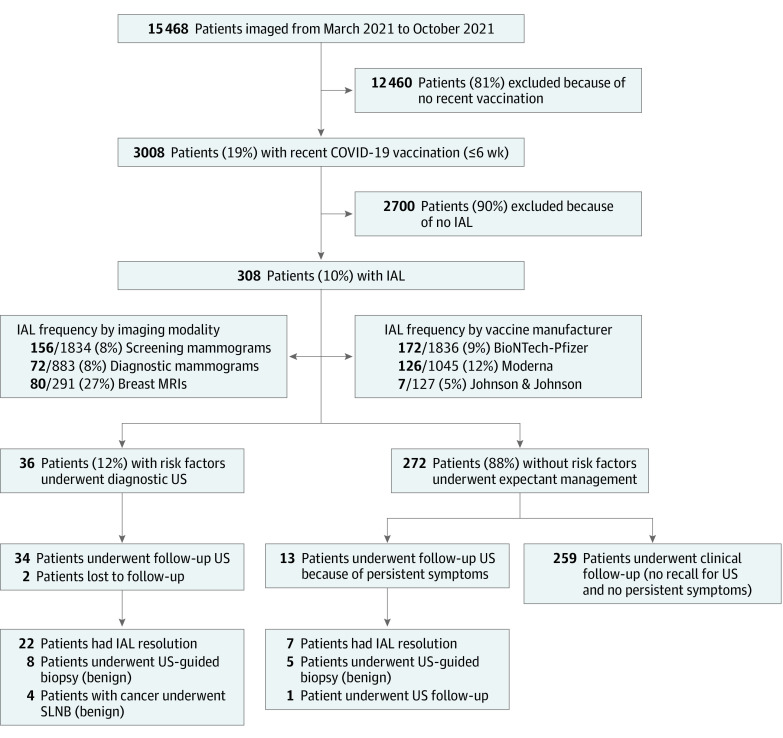
Flowchart of Study Cohort IAL indicates ipsilateral axillary lymphadenopathy; MRI, magnetic resonance imaging; SLNB, sentinel lymph node biopsy; and US, ultrasonography.

The remaining 49 patients with IAL (16%) had recommendations for ultrasonography. A total of 13 symptomatic patients had no risk factors (4%), while 36 patients (12%) had risk factors, including prior breast cancer (23 of 36 [64%]), suspicious ipsilateral imaging results (3 of 36 [8%]), current breast cancer (4 of 36 [11%]), or current regional malignant neoplasm (6 of 36 [17%]) (Table). Two patients with risk factors were lost to follow-up. The remaining 47 patients underwent ultrasonography at a mean (SD) of 8 (4) weeks (34 of 36 patients with risk factors) and 15 (8) weeks (13 of 13 patients without risk factors).

Based on ultrasonography, 29 of 47 patients(62%) had IAL that completely resolved (22 of 34 patients [65%] with risk factors and 7 of 13 patients [54%] without risk factors) (Table and Figure). Based on ultrasonography, the remaining 18 patients (38%) showed persistent IAL with decreased mean (SD) cortical thicknesses (5.2 [1.1] mm at baseline and 3.4 [0.3] mm at follow-up), consistent with resolving reactive lymphadenopathy. Of these 18 patients, 4 had current breast cancer and underwent sentinel lymph node excision (no metastases), 1 without risk factors was recommended for a second, 3-month follow-up ultrasonography, and 13 (8 with risk factors and 5 without risk factors) had biopsies owing to the presence of IAL assessed as BI-RADS (Breast Imaging–Reporting and Data System) 4A at follow-up (11 of 13 [85%]) or patient preference (2 of 13 [15%]). All biopsies showed benign reactive lymphoid hyperplasia.

## Discussion

With more than 70% of US adults receiving the COVID-19 vaccine, evidence-based guidelines for postvaccination IAL are necessary. Although the reported frequency of IAL varies (3%-44%), our data on the frequency of IAL (10%) are similar to those reported for patients in clinical trials (14%).^1,2,3,4,6^

Our study provides a timely assessment of the management and outcome of IAL after COVID-19 vaccination. Of 47 patients with IAL undergoing ultrasonography, 62% had complete IAL resolution, including 22 patients with risk factors. Of patients without risk factors, very few (13 of 308 [4%]) had persistent symptoms requiring ultrasonography or biopsy. Of all patients, including those eventually undergoing biopsy (13 of 308 [4%]), none had malignant neoplasms. However, follow-up for patients with risk factors is important to identify the small number of malignant neoplasms reported by other authors.^2,5,6^

The major limitation of this study is the single-institution design. Further multi-institutional assessments would clarify the patterns and outcomes of postvaccination IAL. Our study shows that IAL after COVID-19 vaccination is frequently a benign reactive finding, supporting expert society guidelines that individualize management approaches tailored to patients’ risk factors, symptoms, and imaging findings.

## References

[1] Robinson KA, Maimone S, Gococo-Benore DA, Li Z, Advani PP, Chumsri S. Incidence of axillary adenopathy in breast imaging after COVID-19 vaccination. JAMA Oncol. 2021;7(9):1395-1397. doi:10.1001/jamaoncol.2021.3127 34292295 PMC8299355

[2] Becker AS, Perez-Johnston R, Chikarmane SA, . Multidisciplinary recommendations regarding post-vaccine adenopathy and radiologic imaging: *Radiology* scientific expert panel. Radiology. 2021;300(2):E323-E327. doi:10.1148/radiol.2021210436 33625298 PMC7909071

[3] Igual-Rouilleault AC, Soriano I, Quan PL, Fernández-Montero A, Elizalde A, Pina L. Unilateral axillary adenopathy induced by COVID-19 vaccine: US follow-up evaluation. Eur Radiol. 2022;32(5):3199-3206. doi:10.1007/s00330-021-08309-7 34655312 PMC8520081

[4] Grimm L, Srini A, Dontchos B. Revised SBI recommendations for the management of axillary adenopathy in patients with recent COVID-19 vaccination. 2022. Society of Breast Imaging. Accessed April 6, 2022. https://www.sbi-online.org/Portals/0/Position Statements/2022/SBI-recommendations-for-managing-axillary-adenopathy-post-COVID-vaccination_updatedFeb2022.pdf

[5] Schiaffino S, Pinker K, Magni V, . Axillary lymphadenopathy at the time of COVID-19 vaccination: ten recommendations from the European Society of Breast Imaging (EUSOBI). Insights Imaging. 2021;12(1):119. doi:10.1186/s13244-021-01062-x 34417642 PMC8378785

[6] Lehman CD, D’Alessandro HA, Mendoza DP, Succi MD, Kambadakone A, Lamb LR. Unilateral lymphadenopathy after COVID-19 vaccination: a practical management plan for radiologists across specialties. J Am Coll Radiol. 2021;18(6):843-852. doi:10.1016/j.jacr.2021.03.001 33713605 PMC7931722

